# Supplementary material for: Housing environment bilaterally alters transcriptomic profile in the rat hippocampal CA1 region
Source: PLoS One. 2025 Dec 4;20(12):e0338190. doi: 10.1371/journal.pone.0338190 (PMC12677517; doi:10.1371/journal.pone.0338190)
Supplement: S1 Fig — (PDF) [file pone.0338190.s001.pdf]

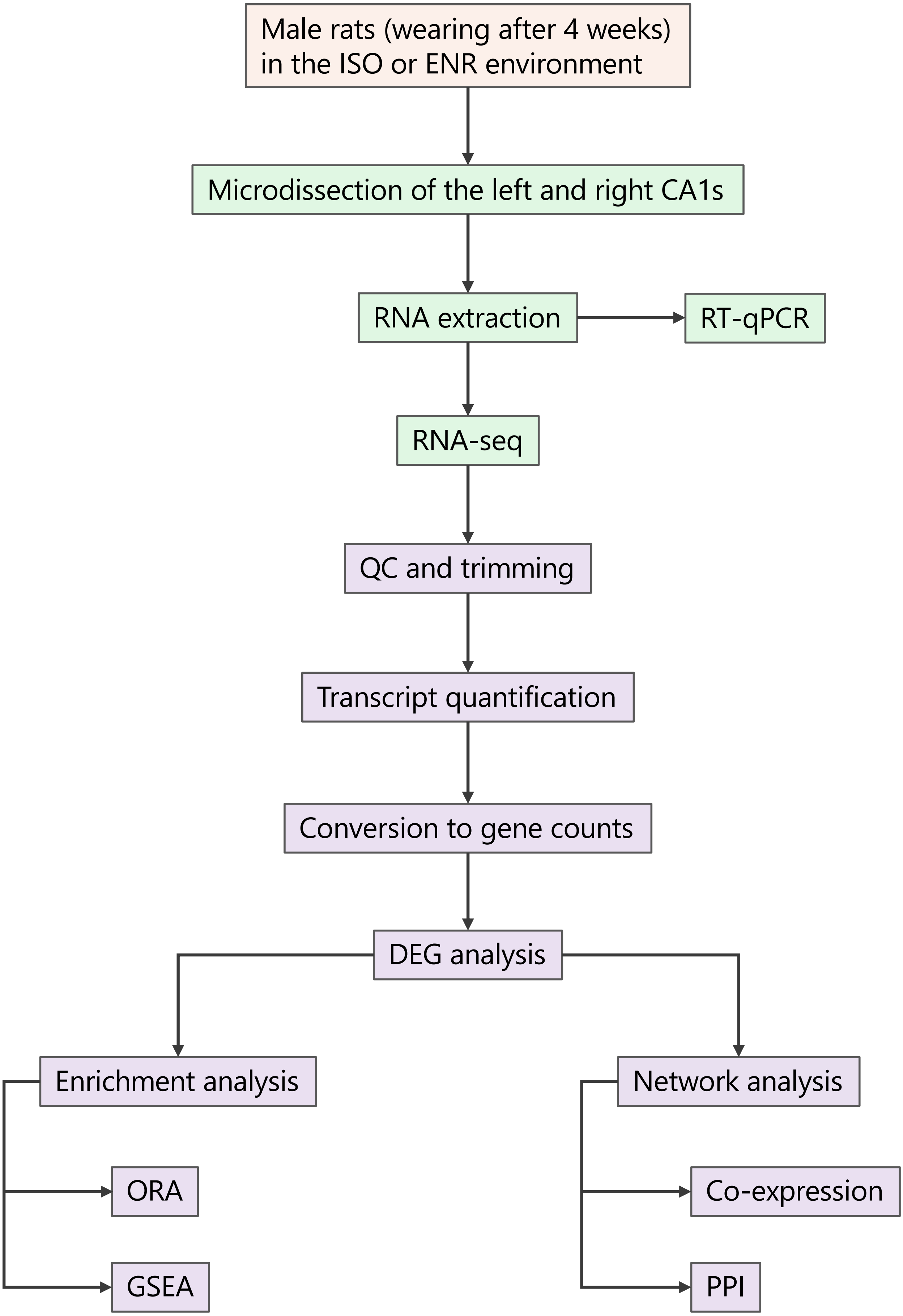

S1 Fig. Brief workflow chart of this study.
ISO: isolated. ENR: enriched. DEG: differentially expressed gene analysis. ORA: over-representation analysis. GSEA: gene set enrichment analysis. PPI: protein-protein interaction.
