# Supplementary material for: Housing environment bilaterally alters transcriptomic profile in the rat hippocampal CA1 region
Source: PLoS One. 2025 Dec 4;20(12):e0338190. doi: 10.1371/journal.pone.0338190 (PMC12677517; doi:10.1371/journal.pone.0338190)
Supplement: S2 Fig — (PDF) [file pone.0338190.s002.pdf]

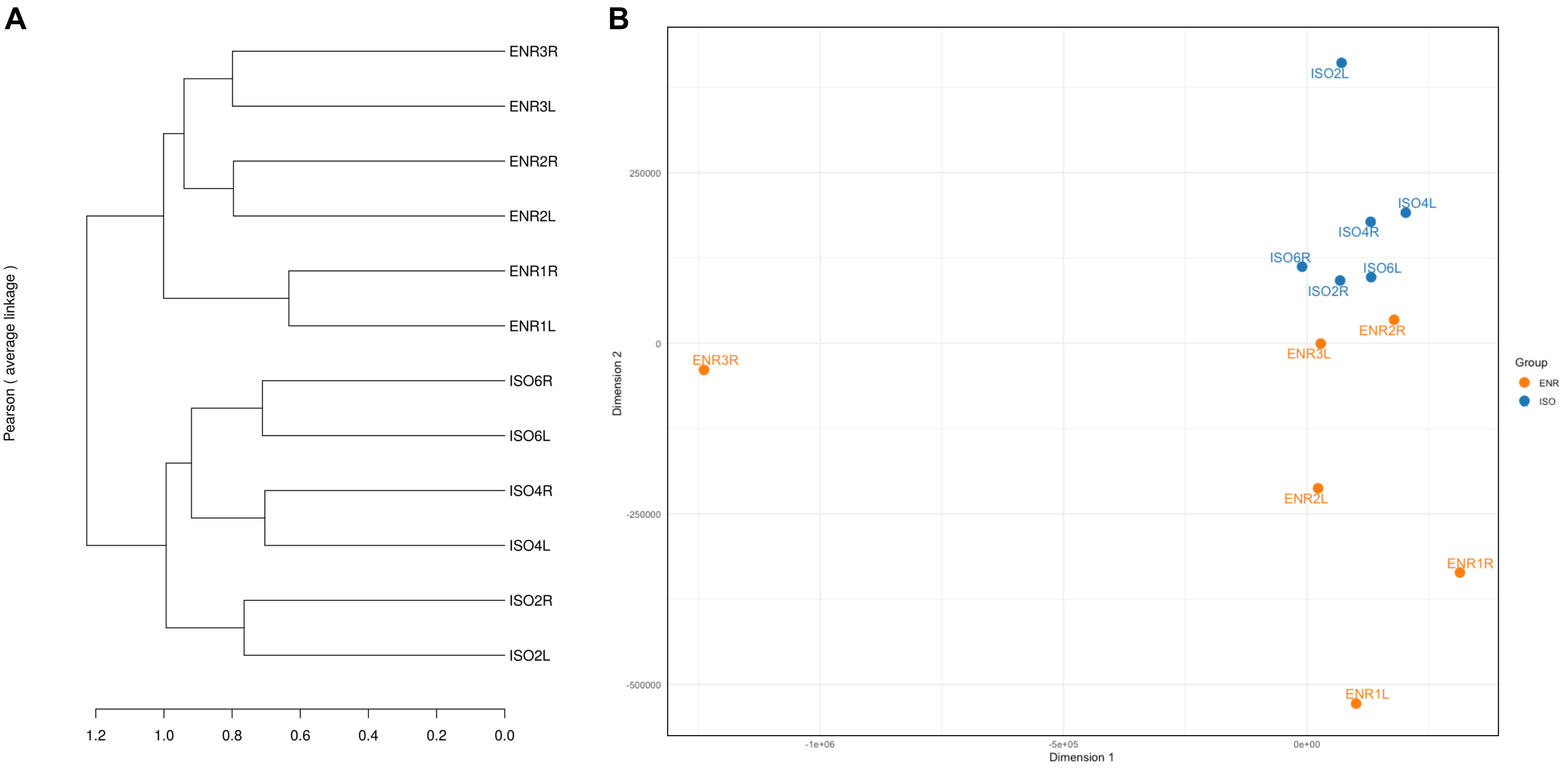

S2 Fig. Multivariate analyses using the TPM-normalized gene count data.

A. Dendrogram of hierarchical clustering result showing the similarity of the samples using the Pearson's correlation method among the normalized gene count data. ISO: isolated condition, ENR: enriched condition, L: left CA1, R: right CA1. **B.** Multi-dimensional scaling plot visualizing the sample similarities and differences. ENR and ISO groups are colored orange and blue, respectively.
