# Supplementary material for: Housing environment bilaterally alters transcriptomic profile in the rat hippocampal CA1 region
Source: PLoS One. 2025 Dec 4;20(12):e0338190. doi: 10.1371/journal.pone.0338190 (PMC12677517; doi:10.1371/journal.pone.0338190)
Supplement: S3 Fig — (PDF) [file pone.0338190.s003.pdf]

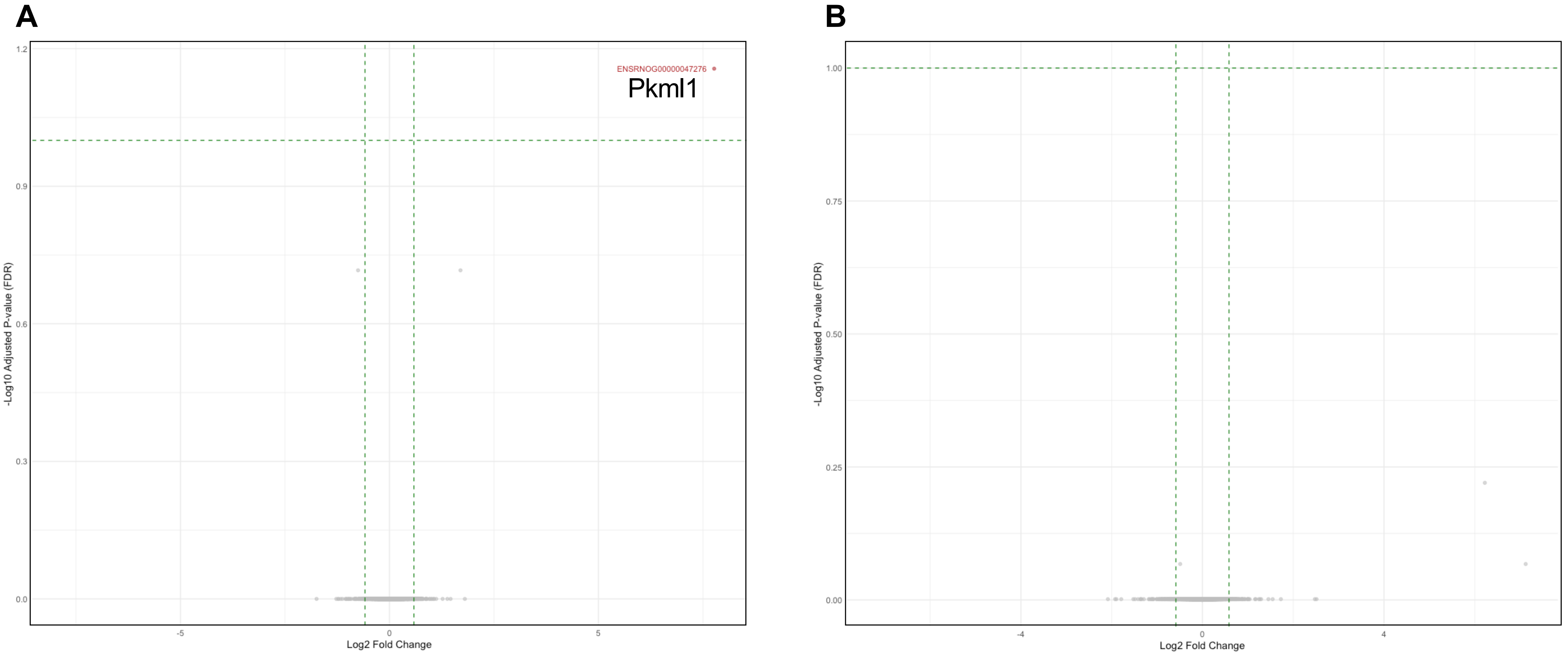

S3 Fig. Volcano plot visualizations of the left–right comparison.

FDR: false discovery rate, FC: fold-change. The thresholds ($\log_2 FC = \pm\log_2 1.5$ and FDR = 0.1) are visualized with green dashed lines. **A.** ISO condition. **B.** ENR condition.
