# Supplementary material for: Housing environment bilaterally alters transcriptomic profile in the rat hippocampal CA1 region
Source: PLoS One. 2025 Dec 4;20(12):e0338190. doi: 10.1371/journal.pone.0338190 (PMC12677517; doi:10.1371/journal.pone.0338190)
Supplement: S4 Fig — (PDF) [file pone.0338190.s004.pdf]

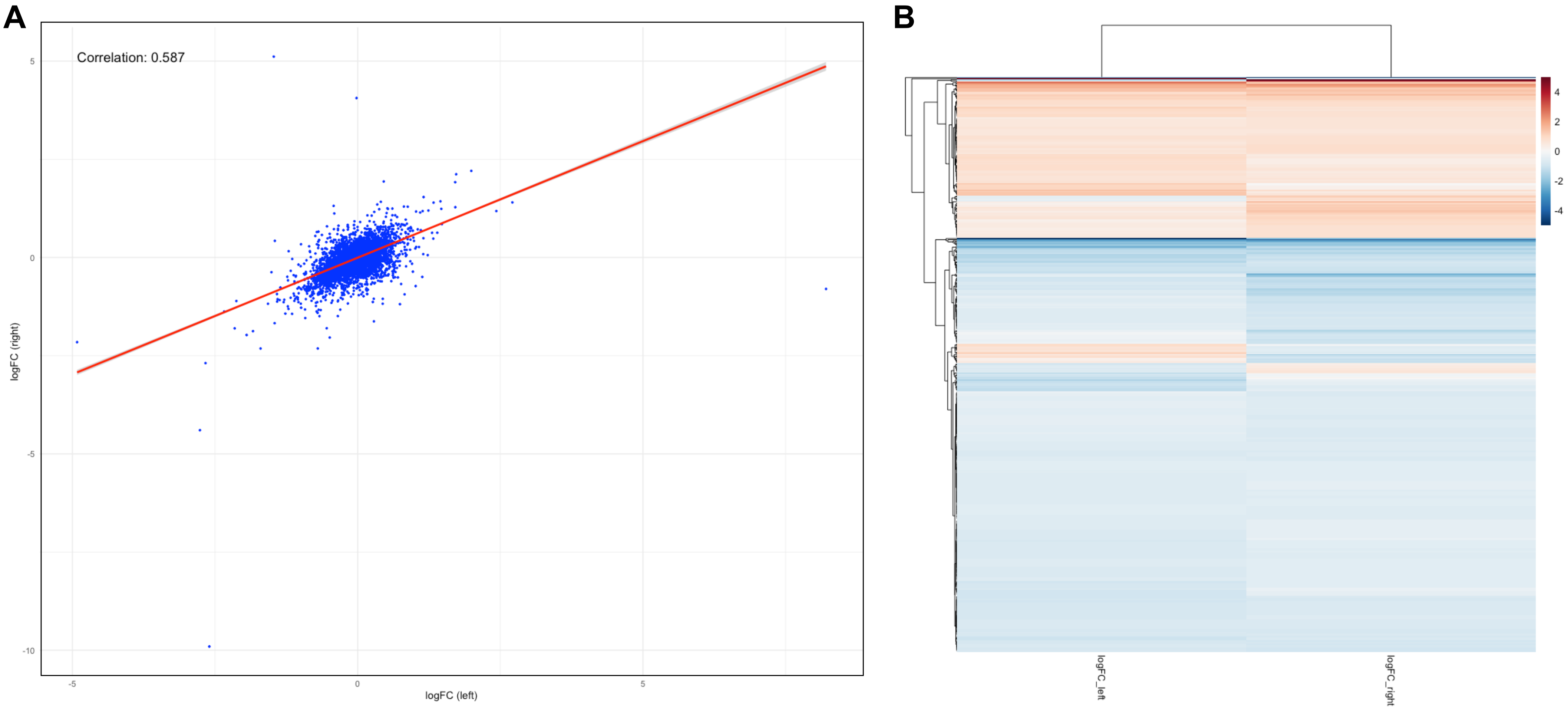

S4 Fig. Correlation plots focusing on the log_2FC values calculated using edgeR.

A. Scatter plot of the log_2FC values for the environmental comparison in the left and right CA1. **B.** Heatmap of the remarkable log_2FC values for the environmental comparison in the left and right CA1.
