# Supplementary material for: Housing environment bilaterally alters transcriptomic profile in the rat hippocampal CA1 region
Source: PLoS One. 2025 Dec 4;20(12):e0338190. doi: 10.1371/journal.pone.0338190 (PMC12677517; doi:10.1371/journal.pone.0338190)
Supplement: S7 Fig — (PDF) [file pone.0338190.s007.pdf]

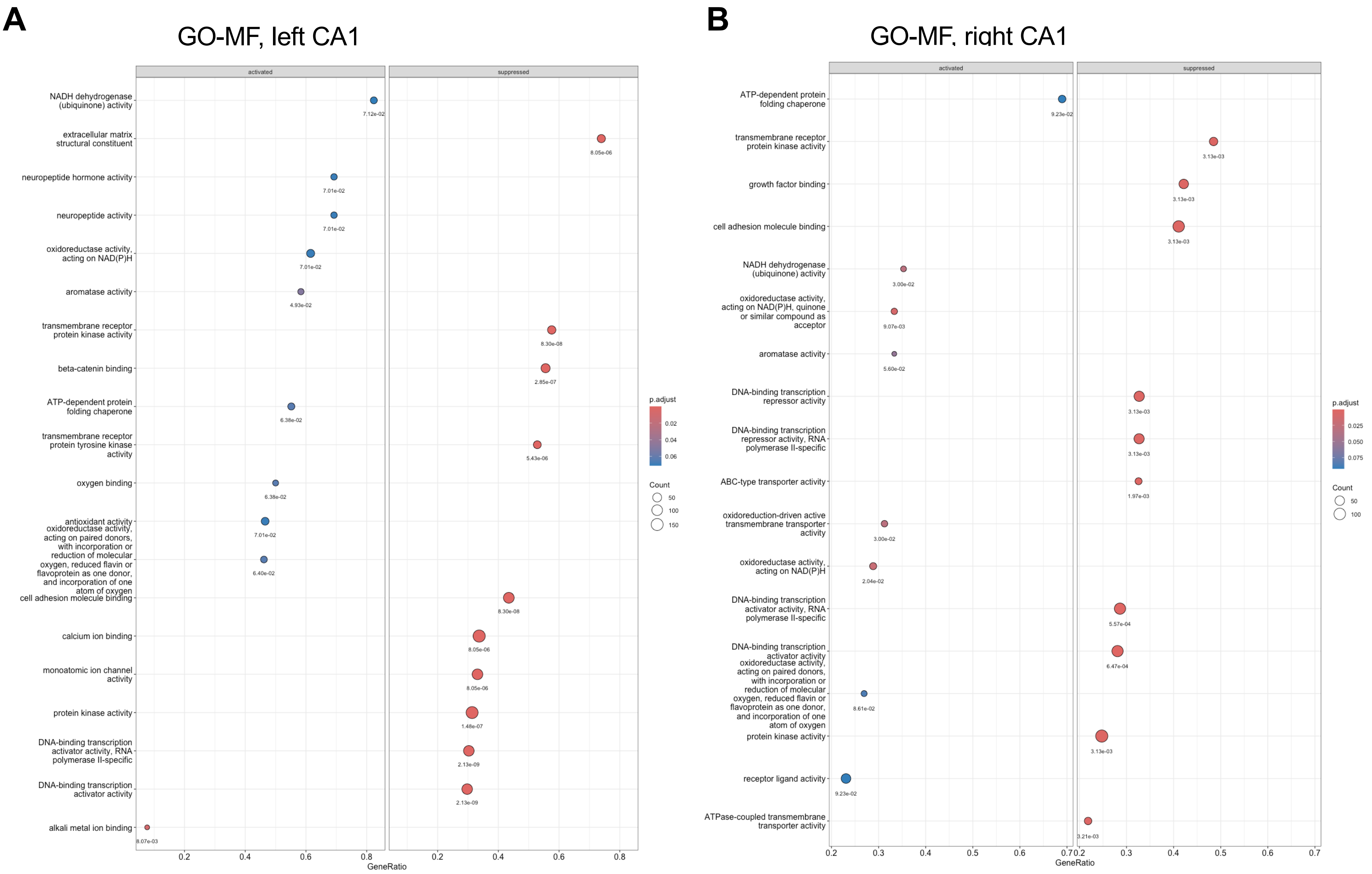

S7 Fig. GSEA for the environmental comparisons in the left and right CA1 regions using the GO-MF database.
A. Dot plot in the left ISO-ENR. **B.** Dot plot in the right ISO-ENR.
