# Supplementary material for: Housing environment bilaterally alters transcriptomic profile in the rat hippocampal CA1 region
Source: PLoS One. 2025 Dec 4;20(12):e0338190. doi: 10.1371/journal.pone.0338190 (PMC12677517; doi:10.1371/journal.pone.0338190)
Supplement: Attachment — Submitted filename: review1.docx [file pone.0338190.s018.docx]

Major revision

The current manuscript mainly involves the environmental regulation of hippocampal lateralization, but there are several problems that limit its scientific nature, and major modifications are recommended.

1. In the abstract, it is recommended to introduce the effects of environment on neuroanatomy and transcriptome in detail and compare with the existing research results. Especially on the impact of the environment on the left and right CA1 regions, it is recommended to increase the relevant literature support.

2.For data analysis, especially the description of statistical methods is relatively simple, and the lack of specific explanation for the selection of key parameters, it is recommended to describe the statistical methods of data analysis in detail.

3.There is a lack of in-depth explanation in the results, especially for the 'no DEG detected ' part; it is recommended to increase the discussion to explain the possible reasons.

4. It is suggested to discuss possible mechanisms, such as differences in neuroanatomy and function between left and right CA1, or how environmental stimuli affect gene expression through stress response.

5. It is recommended that the function and underlying mechanisms of each key gene be discussed in detail, including those genes that may not be expressed significantly in the DEG analysis, as they may play a role in environmental stress.

6.The limitations of the article were discussed in more detail, especially the influence of gender differences on the heterogeneity of left and right CA1.

7.Slightly simplify some complex sentences to ensure that they are concise and clear.

8.Improve the logic of the article and avoid repeated statements.

9. The references cited in this article are not sufficient, and there is a lack of in-depth comparative discussion. Background and methodology also require further literature support. Some related research should be cited:

1. Zn‐DHM Nanozymes Enhance Muscle Regeneration Through ROS Scavenging and Macrophage Polarization in Volumetric Muscle Loss Revealed by Single‐Cell Profiling

2. An injectable multi-functional composite bioactive hydrogel for bone regeneration via immunoregulatory and osteogenesis effects

3. Single-cell RNA sequencing and immune microenvironment analysis reveal PLOD2-driven malignant transformation in cervical cancer

4. Single-cell analysis unveils cell subtypes of acral melanoma cells at the early and late differentiation stages

5. The cellular signaling crosstalk between memory B cells and tumor cells in nasopharyngeal carcinoma cannot be overlooked: Their involvement in tumor progression and treatment strategy is significant

6. Single-Cell RNA-Sequencing Integration Analysis Revealed Immune Cell Heterogeneity in Five Human Autoimmune Diseases

7. Prognostic Value of Tumor-microenvironment-associated Genes in Ovarian Cancer
