# Supplementary material for: Housing environment bilaterally alters transcriptomic profile in the rat hippocampal CA1 region
Source: PLoS One. 2025 Dec 4;20(12):e0338190. doi: 10.1371/journal.pone.0338190 (PMC12677517; doi:10.1371/journal.pone.0338190)
Supplement: Attachment — Submitted filename: Response to Reviewers.docx [file pone.0338190.s020.docx]

**Response to Reviewers (PONE-D-25-08336)**

To Reviewer #1

*1. In the abstract, it is recommended to introduce the effects of environment on neuroanatomy and transcriptome in detail and compare with the existing research results. Especially on the impact of the environment on the left and right CA1 regions, it is recommended to increase the relevant literature support.*

Response:

Thank you for this helpful suggestion. After careful consideration, we decided that revising the Introduction section of the manuscript would be more effective than modifying the Abstract. Specifically, we have rewritten the paragraph introducing the effects of the environment on neuroanatomy and the transcriptome (lines 72–92) to draw more support from the relevant literature. Additionally, we have increased the number of citations to strengthen the supporting evidence from previous studies, increasing the total from 36 to 65.

----------

*2. For data analysis, especially the description of statistical methods is relatively simple, and the lack of specific explanation for the selection of key parameters, it is recommended to describe the statistical methods of data analysis in detail.*

Response:

Thank you for your comments. Given that the specific part lacking details was not specified, we have added further explanations to enhance the description of the analytical methods in the Materials and Methods section of the revised manuscript (lines 156–161, 180–184, 193–198, and 206–215).

----------

*3. There is a lack of in-depth explanation in the results, especially for the 'no DEG detected ' part; it is recommended to increase the discussion to explain the possible reasons.*

Response:

Thank you for your valuable comments. In this revised manuscript, we adopted less stringent thresholds in response to Comment 1 from Reviewer #2. We identified one differentially expressed gene (DEG) in the left–right comparison within the ISO group. Although no DEGs were detected in the ENR group, even with the relaxed thresholds, we have added a discussion of the possible reasons why the present study did not reveal notable hemispheric differences in gene expression (lines 554–565).

----------

*4. It is suggested to discuss possible mechanisms, such as differences in neuroanatomy and function between left and right CA1, or how environmental stimuli affect gene expression through stress response.*

Response:

Thank you for the important suggestion. We agree with your suggestion. Accordingly, we have substantially revised the Discussion section to introduce the possible mechanisms through which living environments may contribute to hippocampal asymmetry. For example, in lines 482–490, we described the proposed role of transcriptomic differences in generating hippocampal asymmetry: “Collectively, these ORA and GSEA results consistently demonstrate that distinct molecular mechanisms underpin the effects of environmental treatments on the left and right hippocampal CA1 regions. While the left CA1 region facilitates coordinated metabolic activity and structural remodeling, the right CA1 region selectively activates endogenous gene regulatory mechanisms, potentially suppressing experience-driven neural plasticity in response to enriched environments. These findings highlight qualitative differences in the molecular strategies employed by each hemisphere in response to environmental stimuli, offering a detailed perspective on functional asymmetry and localized response specificity in the hippocampus.”

----------

*5. It is recommended that the function and underlying mechanisms of each key gene be discussed in detail, including those genes that may not be expressed significantly in the DEG analysis, as they may play a role in environmental stress.*

Response:

Thank you for your recommendations. In the revised manuscript, we have expanded the discussion of specific DEGs to strengthen the interpretation of our findings. As mentioned previously (in response to comment 3), the use of a new FDR threshold revealed numerous candidate DEGs that warranted further discussion in the context of hippocampal asymmetry and environmental influences on gene expression profiles. We believe that the current revision, particularly the updated discussion on the role of the environment (e.g., lines 491–534), addresses the concerns raised in the previous version of our manuscript.

----------

*6. The limitations of the article were discussed in more detail, especially the influence of gender differences on the heterogeneity of left and right CA1.*

Response:

Thank you for the suggestion. We have explained the limitations of our study in more detail, including the potential influence of sex differences on hippocampal laterality. Particularly, we introduced a new subsection titled “Limitations and perspectives” in the Discussion section to clarify how our findings may contribute to future research and which aspects remain inconclusive based on the current study.

----------

*7. Slightly simplify some complex sentences to ensure that they are concise and clear.*

*8. Improve the logic of the article and avoid repeated statements.*

Response:

Thank you for your suggestions regarding the structure of the manuscript. Comments 7 and 8 reflected a similar perspective. To improve the logical flow and eliminate redundant statements, we have revised the manuscript with the assistance of a language-polishing service. This also helped to enhance the overall quality of the writing. We hope that these revisions have addressed your concerns.

----------

*9. The references cited in this article are not sufficient, and there is a lack of in-depth comparative discussion. Background and methodology also require further literature support. Some related research should be cited:*

*1. Zn‐DHM Nanozymes Enhance Muscle Regeneration Through ROS Scavenging and Macrophage Polarization in Volumetric Muscle Loss Revealed by Single‐Cell Profiling*

*2. An injectable multi-functional composite bioactive hydrogel for bone regeneration via immunoregulatory and osteogenesis effects*

*3. Single-cell RNA sequencing and immune microenvironment analysis reveal PLOD2-driven malignant transformation in cervical cancer*

*4. Single-cell analysis unveils cell subtypes of acral melanoma cells at the early and late differentiation stages*

*5. The cellular signaling crosstalk between memory B cells and tumor cells in nasopharyngeal carcinoma cannot be overlooked: Their involvement in tumor progression and treatment strategy is significant*

*6. Single-Cell RNA-Sequencing Integration Analysis Revealed Immune Cell Heterogeneity in Five Human Autoimmune Diseases*

*7. Prognostic Value of Tumor-microenvironment-associated Genes in Ovarian Cancer*

The suggested references are not directly relevant to this manuscript. Accordingly, we believe that adding the citations to the manuscript would be inappropriate. Hence, we have decided not to include the suggested references in the manuscript. Thank you for your comments.

----------

To Reviewer #2

*1. The authors set a relatively strict DEG screening standard (log2FC > ±0.58, FDR < 0.05), resulting in a small number of differentially expressed genes being screened out: only 13 on the right CA1 and only 105 on the left CA1. This number is insufficient to support an in-depth interpretation of the transcriptional response of complex brain regions under environmental intervention. It is recommended to supplement the use of a more lenient threshold (such as FDR < 0.1 or p < 0.05) for trend differential expression analysis and provide MA plots, rank plots, or a complete list of gene expression logFC to show the overall expression trend or clearly explain the statistical power and limitations of the current results, avoiding over-interpretation of the results.*

Response:

We appreciate your valuable comment and agree fully with your suggestions. Accordingly, we adopted a less stringent FDR threshold (relaxed from 0.05 to 0.1). This modification increased the number of environment-induced DEGs, thereby enhancing the informativeness of the enrichment analyses, especially in the over-representation analyses. As a result of this improvement in the DEG analyses, we strengthened the Discussion section of the revised manuscript. Additionally, the complete expression dataset is provided in the Supplementary Information. We have also addressed the issues of sample size and statistical power in the Limitations and Perspectives subsection of the Discussion (lines 554–565).

----------

*2. Some of the tools used in the current study (such as Salmon v0.8.1, GeneMANIA, and org.Rn.eg.db v3.20) are significantly outdated, which may affect the accuracy and reproducibility of the analysis. It is recommended to upgrade Salmon to v1.10 or above, supplement bias correction and other functions, and annotate the used Ensembl version, or use the latest GENCODE or Ensembl v110. In the GSEA part, it is recommended to introduce the MSigDB database (through the msigdbr package) to enhance the breadth and interpretability of the GO enrichment results. For co-expression analysis, it is recommended to combine the STRING database or use more robust analysis tools such as WGCNA.*

Response:

Thank you for your suggestion regarding the version of the tools used. First, we reanalyzed the sequencing data using the latest version of Salmon (v1.10.3), along with the Ensembl release 113 reference dataset for mRatBN7.2 cDNA assembly and its corresponding genomic annotation. We also confirmed that the validateMapping option, which improves the accuracy of transcript quantification, was enabled during salmon analysis. Second, we incorporated MSigDB and its R package, msigdbr, to further characterize the GSEA results. Specifically, we used the C2 category gene sets from the KEGG and Reactome Pathway collections in this revision. Third, we performed protein–protein interaction analyses using the STRING database to highlight the relationships among environment-induced DEGs at the post-transcriptional level. Based on your suggestion, we are confident that the scientific value of this study has been further enhanced.

----------

*3. The current discussion provides relatively brief mechanistic explanations for several key findings and fails to fully integrate existing research for in-depth exploration. It is recommended to deeply discuss the functions of key genes (such as Fos, Cdkl5, Grin2b, etc.) in neural activity and analyze the biological significance of pathways such as synaptic transmission and cell connections based on GSEA enrichment pathways. In the conclusion section, the language should be more cautious, clearly stating that the study is a preliminary exploration.*

Response:

We appreciate your recommendations regarding the Discussion section. As the present study was improved by adopting new DEG thresholds, we have made extensive revisions to this section. Additional mentions and discussions of specific DEGs, including *Fos*, *Cdkl5*, and *Grin2b*, were incorporated based on the combined results of enrichment and network analyses (lines 491–526). Furthermore, by adding the Limitations and Perspectives subsection to the Discussion, we revised the manuscript to adopt a more cautious tone and clearly acknowledged that the current study represents a preliminary exploration.

We appreciate your helpful suggestions, which have significantly contributed to improving the clarity and scientific depth of our manuscript.

----------

*4. With only three biological replicates per group, it may not be sufficient to support the detection of DEGs in some inter-group comparisons (such as left and right CA1). It is recommended to supplement statistical power assessment or point out this limitation in the discussion.*

Response:

Thank you for this valuable suggestion. As noted in our response to Comment 3 from Reviewer#1, by applying the new FDR threshold, we identified one DEG in the left–right comparison within the ISO group. Although no DEGs were found in the ENR group, even with less stringent thresholds, we have discussed the possible reasons why our study did not observe notable DEGs between the hemispheres (lines 491–553). As previously addressed in our response to Comment 1 regarding statistical power, we discuss the sample size and its implications for statistical power in the Limitations and Perspectives subsection (lines 554–565).

----------

*5. This study has potential value, but the current data support and argumentation process are not yet complete. It is recommended to deepen the expression analysis, clarify the analysis methods and versions and the scope and limitations of the results, and strengthen the biological interpretation and mechanism exploration.*

Response:

We appreciate your comment regarding the potential value and current limitations of the interpretation of our study. In response, we have made several key revisions to deepen our biological insight and enhance the rigor of our transcriptomic analyses. First, we conducted overrepresentation analyses to further characterize the unilateral DEGs identified in the environmental comparisons. Second, following your recommendation, we performed protein–protein interaction network analyses using the STRING database. Third, we performed an additional GSEA using MSigDB gene sets to supplement the interpretation of gene expression signatures associated with housing conditions. We believe that these modifications substantially strengthened the transcriptomic analysis and biological interpretation in the revised manuscript. The Materials and Methods section has also been updated to include more detailed information on the versions of software and tools used, as well as specific analytical parameters. Furthermore, we have added a new subsection titled “Limitations and perspectives” to the Discussion section to clarify both the implications of our findings for future research and the conclusions that cannot be drawn from the present data.

----------

*6. It is recommended to discuss in the discussion the possible influence of gender factors on the lateralized expression of CA1 and its necessity in future research. In addition, there are some minor suggestions, such as adding an experimental flowchart, making the chart annotations clearer, unifying the abbreviations of terms, and using language polishing services to improve the quality of English.*

Response:

Thank you for the suggestion. In response, we have added a discussion on the possible influence of sex-related factors on hippocampal lateralization and emphasized its importance in future research (lines 536–544). We have prepared the brief workflow chart of this study in S1 Fig. Additionally, with the assistance of a professional language editing service, we have improved the overall clarity and readability of the manuscript, revised the figure legends, and ensured consistency in the use of abbreviations throughout the text.

*The references cited in this article are not sufficient, and there is a lack of in-depth comparative discussion. Background and methodology also require further literature support. Some related research should be cited:*

*10.1111/cpr.13376*

*10.18240/ijo.2019.08.04*

*10.34133/research.0183*

*10.34133/research.0134*

As noted in our response to Comment 9 from Reviewer #1, the suggested citations fall outside the scope of our study. Therefore, we respectfully chose not to include them and appreciate your understanding.

----------
