# Supplementary material for: Housing environment bilaterally alters transcriptomic profile in the rat hippocampal CA1 region
Source: PLoS One. 2025 Dec 4;20(12):e0338190. doi: 10.1371/journal.pone.0338190 (PMC12677517; doi:10.1371/journal.pone.0338190)
Supplement: Attachment — Submitted filename: Response_to_Reviewers_auresp_2.docx [file pone.0338190.s021.docx]

**Response to Reviewers (PONE-D-25-08336R1)**

To Reviewer #3

*The manuscript "Housing environment bilaterally alters transcriptomic profile in the rat hippocampal CA1 region" is an interesting one and has been conducted scientifically. The authors have addressed the suggestions of the reviewers.*

**Response:**

We sincerely thank Reviewer #3 for the positive and encouraging feedback on our work.

------

To Reviewer #4

*The study analyzes data obtained from only three animals per group, which limits the strength of the conclusions. It does not consider the cellular composition of the CA1 region or how environmental factors might impact this composition and, in turn, gene expression. Greater integration with existing literature is needed to contextualize the findings, as was requested by previous reviewers. This has not been sufficiently addressed.*

*e.g from reviewer: 4. It is suggested to discuss possible mechanisms, such as differences in neuroanatomy and function between left and right CA1*

**Response:**

We sincerely thank the reviewer for the thoughtful and constructive feedback. We have carefully revised the manuscript to address all the concerns raised, particularly by improving the integration with existing literature and providing a clearer discussion of the study’s limitations.

1. Sample size:

We fully agree that the small sample size represents a critical limitation of our study. In the first-round revised manuscript, we have already acknowledged this issue in the “Limitations and perspectives” section, emphasizing the limited statistical power and the potential risk of both false negatives and false positives (Lines 585–596 in the latest version). We also believe that our previous statement—highlighting that “large-scale studies with adequate statistical power are essential for robust and reliable identification of lateralized and environment-induced gene expression profiles” (Lines 594–596) —adequately addresses this concern.

2. Cellular composition:

We appreciate this important point. We have added a new paragraph to the “Limitations and perspectives” section noting that our bulk RNA-seq cannot distinguish cell-type-specific transcriptional changes from possible shifts in cellular composition. We also indicate that future studies employing single-cell RNA sequencing (scRNA-seq) will be essential to clarify these aspects (Lines 603–608).

3. Integration with existing literature (neuroanatomy and function):

We have expanded the Discussion to provide stronger integration with relevant neuroanatomical and functional studies.

- Neuroanatomy: In the revised Discussion (Lines 512–521), we now link our molecular findings on metabolic activity to neuroanatomical features, suggesting that the left CA1 may utilize higher metabolic activity to fuel structural plasticity, such as the remodeling of synaptic ultrastructure.
- Function: In the same paragraph, we further connect our findings with prior reports on hippocampal functional asymmetry by proposing that the observed molecular asymmetry may represent a potential “molecular substrate” for the lateralized gamma oscillations previously described (Line 517–519).

We hope these revisions adequately address the reviewer’s comments and improve the clarity and contextual depth of the manuscript.

------

*It is commendable that the Limitations section acknowledges the flaws in the study, demonstrating an awareness of the study’s constraints.*

**Response:**

We sincerely thank the reviewer for the positive feedback on the Limitations section. We greatly appreciate the recognition of our efforts to acknowledge and transparently discuss the constraints of this study.

------

*While gene validation was performed using qPCR, the rationale as to the selection of two genes compared to all identified is not addressed. Were others analysed but not validated? There is no figure 2C. The number of animals included in this validation is also not specified. It is also unclear whether the same samples were used or if different ones were analyzed. These issues should be explicitly addressed in the Limitations section.*

**Response:**

We thank the reviewer for these valuable comments. The figure numbering has been corrected accordingly (there is no Figure 2C in the second-round revised version). Regarding the RT-qPCR validation, we have clarified in the Materials and Methods section that two representative DEGs were selected solely to validate the RNA-seq results (Lines 166–169). The same RNA samples used for RNA sequencing were employed for qPCR, with n = 3 per condition, consistent with the RNA-seq analysis (Lines 169–170). As the purpose of this experiment was to confirm the consistency of expression trends rather than to provide an independent replication, we believe that this clarification in the Methods section appropriately addresses the reviewer’s concern, and therefore it was not added to the Limitations section.

------

*The rationale for each statistical approach should be clearly articulated. It is important to explain why each test was chosen and why it is appropriate for such small sample sizes. Without this justification, the robustness of the statistical conclusions remains uncertain.*

**Response:**

We appreciate the reviewer’s insightful comment. We agree that clearly articulating the rationale for each statistical method enhances confidence in the analytical robustness. Accordingly, we have revised the Materials and Methods section to include concise explanations of the statistical approaches employed.

Specifically, we now state that edgeR was chosen because it uses an empirical Bayes framework to stabilize dispersion estimates across genes, making it particularly suitable for RNA-seq data with limited biological replicates (Lines 144–146). We also added that Welch’s *t*-test was applied for RT-qPCR analysis to account for potential heterogeneity of variance between groups, providing more reliable inference with small sample sizes (Lines 176–178). These additions clarify that the statistical methods were selected with careful consideration of both the small sample size and the characteristics of data, thereby ensuring an appropriate and robust analytical framework.

------

To Reviewer #5

*Major Points:*

*1. As other reviewers may have noted, the interpretation of the results is insufficient. The Results section, in particular, merely describes the findings without any interpretation, making it unclear what conclusions can be drawn from each experiment. The authors should interpret their findings within the Results section or significantly expand the Discussion to connect their data to biological meaning.*

**Response:**

We agree with the reviewer that the Results section should primarily present the findings, whereas the Discussion section should provide their interpretation. We have maintained this standard IMRaD structure. However, we appreciate the reviewer's observation that the biological implications of each analysis were not immediately clear. To enhance readability and better guide the reader, we have revised the Results section to include concise summative statements at the end of key subsections: **DEG analysis and RT-qPCR** (Lines 284–287), **ORA of bilateral DEGs in the environmental comparison** (Lines 353–356) and **GSEA in the environmental comparison** (Lines 450–454). These statements succinctly summarize the direct outcomes of each analysis without introducing additional interpretation, which is reserved for the Discussion. We believe that these revisions make the presentation of the results substantially clearer, as suggested.

------

*2. The authors conclude that there are significant hemispheric differences based on their results. However, it is generally expected that some differences, including experimental noise, will emerge in any omics analysis. This reviewer thinks that a more appropriate conclusion from these data would be that there are few, if any, meaningful differences between the hemispheres. The authors must define the criteria under which they would conclude a lack of hemispheric difference and provide stronger evidence that the observed differences are biologically significant, not just experimental noise or statistical artifacts.*

**Response:**

We sincerely thank the reviewer for this critical and insightful comment, which addresses the core interpretation of our findings. We fully understand the reviewer’s skepticism: given that the direct left–right comparison yielded 0–1 DEGs, why do we conclude there is a significant hemispheric difference?

Our central claim does not concern baseline expression differences, but rather a qualitative divergence in the transcriptomic response (or “reactivity”) to environmental stimuli. The reviewer is correct that this “functional” asymmetry, derived from a limited sample size (n = 3), could potentially reflect a statistical artifact. However, we contend that the observed difference is biologically meaningful for the following reasons:

- Coherent Biological Themes: The observed differences are not random noise. Both ORA and GSEA revealed distinct, biologically coherent themes for each hemisphere—left: synaptic function, memory, and metabolism; right: RNA metabolism and transcriptional regulation. It is highly unlikely that random variation would consistently generate such distinct, functionally cohesive pathway signatures.
- Integration with External Evidence: As also suggested by other comments (e.g., Reviewer #1, Comment 4), we have explicitly linked these hemisphere-specific molecular themes to existing functional and anatomical literature. In the Discussion section (Lines 512–521), we now explain how the “metabolic and structural” theme observed in the left CA1 aligns with the concept of synaptic ultrastructure remodeling. Furthermore, we cited Shinohara et al. (2013) to propose that our observed molecular asymmetry may serve as a potential substrate for their reported functional asymmetry. This convergence with independent findings strengthens the argument that our results reflect genuine biological asymmetry rather than experimental noise.

To clarify this key point, we have also added sentences to the Discussion section (Lines 465–472) explicitly acknowledging the high baseline similarity (0–1 DEGs) while justifying our interpretation of a qualitative hemispheric asymmetry in environmental reactivity, grounded in coherent and reproducible biological themes rather than statistical artifacts. Importantly, we also emphasize that this interpretation does not constitute a definitive conclusion but rather a hypothesis that warrants validation in future large-scale studies with greater statistical power in the Limitations section (Lines 585–596).

Finally, in response to the reviewer’s question on criteria, we would have concluded that there was no hemispheric difference if both ORA and GSEA for the left and right (ISO vs. ENR) comparisons had revealed substantial overlap in enriched functional pathways. The fact that these analyses instead identified distinct and non-overlapping biological processes forms the basis for our conclusion. We believe that our revised explanation and explicit acknowledgment of the need for further validation appropriately address the reviewer’s concern.

------

*3. It is inappropriate to assess hemispheric differences based solely on whether a gene passes a specific DEG threshold in one hemisphere but not the other. A gene identified as a DEG in one hemisphere might show a similar trend (i.e., similar direction and magnitude of change) in the opposite hemisphere, even if it does not meet the arbitrary p-value or fold-change cutoff. To substantiate the claim that the changes are truly hemisphere-specific, the authors should, for 150 genes, present bar graphs displaying the expression levels and corresponding p-values for both hemispheres side-by-side. This would provide a more transparent and convincing comparison.*

**Response:**

We fully agree with the reviewer’s fundamental statistical point: assessing hemispheric differences solely by determining whether a gene passes an arbitrary DEG threshold in one hemisphere but not the other is not a robust approach. However, we would like to clarify that this is not the basis of our study’s conclusion. Our interpretation of hemispheric difference is not derived from such a “gene-by-gene” comparison.

Our argument is instead based on higher-level *in silico* functional analyses (ORA and GSEA). Specifically, the complete set of 189 DEGs identified in the left CA1 is enriched for “synaptic function and metabolism,” whereas the 94 DEGs in the right CA1 are associated with “transcriptional and RNA regulation” processes. The significant difference we emphasize lies in these biologically coherent pathway signatures rather than in individual gene-level contrasts.

To directly address the reviewer’s concern that genes might show similar trends across hemispheres, we performed a correlation analysis using the log_2_FC values for all genes between the left and right CA1 regions (S4 Fig). This analysis yielded a Pearson coefficient of *r* =0.587, confirming a moderate overall directional similarity in gene expression changes across hemispheres.

We believe this result actually strengthens our main argument: despite this substantial correlation in overall expression trends, the subset of the most significantly regulated genes on each side diverged into functionally distinct biological themes (synaptic vs. regulatory). This pattern is unlikely to result from a simple thresholding artifact.

Because our interpretation is drawn from pathway-level integration, and the correlation analysis already provides a comprehensive overview of interhemispheric similarity (S4 Fig), we do not believe that adding bar graphs for 150 individual genes would further clarify our central message.

------

*4. This paper used an “Isolation” condition as a control compared to an “Enriched environment.” Since isolation eliminates social communication, which is critical for normal mouse development, it is questionable whether this condition serves as a proper neutral baseline. A group housing condition should have been included as a control group. This would allow for a clearer determination of how transcription becomes altered by enriched environment.*

**Response:**

We thank the reviewer for raising this important point regarding the experimental design. We would like to clarify a crucial aspect of our study’s aim and rationale, as there may be some misunderstanding of our experimental objective.

The reviewer is correct that the “Isolation” (ISO) condition is not a physiologically neutral baseline. However, our study was not intended to use ISO as a “neutral control.” Rather, the objective was to directly compare the transcriptomic effects of two distinct and well-defined environmental conditions: social isolation (ISO) vs. sensorimotor enrichment (ENR). This specific comparison (ISO vs. ENR) was intentionally chosen because it reproduces the exact experimental paradigm used in the foundational functional literature on hemispheric lateralization, upon which our study builds.

Our goal was to identify the molecular substrates that may underlie the functional lateralization (e.g., in hippocampal gamma oscillations, as reported by Shinohara et al., 2013) observed under these same environmental manipulations. Therefore, we believe that our experimental design—which directly parallels and extends prior work in this field—is appropriate and valid for addressing our research question.

------

*Minor Points:*

*1. Although the authors mention the duration of housing in either the isolated or enriched environment in the Discussion, this information is essential for reproducibility and should be clearly stated in the Materials and Methods section.*

**Response:**

We thank the reviewer for this comment. The duration of housing under the isolated or enriched environment is already described in the Materials and Methods section (Lines 113–114: “Hippocampal slices were prepared... 4 weeks after rearing under the respective environmental conditions”).

------

*2. The volcano plots should be annotated with gene names instead of, or in addition to, gene IDs to improve readability and facilitate immediate interpretation.*

**Response:**

We agree with the reviewer that annotating the volcano plots with gene names improves immediate readability. The reviewer is correct that the plots in the original manuscript were labeled only with gene IDs, with the full list provided in S4 Data. As the reviewer suggested, this is not ideal for quick interpretation. We also note that labeling all DEGs would compromise the legibility of the plots.

As a compromise, we have revised Figure 1 to include gene symbol annotations for selected DEGs, particularly those discussed in the Discussion section. This revision highlights the key drivers of the transcriptomic changes at a glance, addressing the reviewer's concern while maintaining the visual clarity of the plots. The complete DEG lists remain available in S4 Data.

------
